# Envelope-dimer epitope 1 (EDE1) antibody (C10) treatment significantly reduces Zika virus replication in the male and female reproductive tracts

**DOI:** 10.1128/jvi.01147-25

**Published:** 2025-08-18

**Authors:** Nathaniel J. Schramm, Martina Kovarova, Shajer Manzoor, Adam S. Cockrell, Rae Ann Spagnuolo, Franck Amblard, Leda Bassit, Raymond F. Schinazi, Ralph S. Baric, Angela Wahl, J. Victor Garcia

**Affiliations:** 1 Division of Infectious Diseases, Center for AIDS Research, University of North Carolina at Chapel Hill 502984, Chapel Hill, North Carolina, USA; 2 Department of Microbiology, University of Alabama at Birmingham 318277, Birmingham, Alabama, USA; 3 Department of Epidemiology, The University of North Carolina at Chapel Hill 154805, Chapel Hill, North Carolina, USA; 4 Center for ViroScience and Cure, Laboratory of Biochemical Pharmacology, Department of Pediatrics, Emory University School of Medicine and Children’s Healthcare of Atlanta 12239, Atlanta, Georgia, USA; The Ohio State University, Columbus, Ohio, USA

**Keywords:** Zika virus, eye, immunodeficient, reproductive tract, neutralizing antibodies, preclinical model, brain, prevention

## Abstract

No effective therapy or vaccine exists to protect against the next Zika virus (ZIKV) outbreak. ZIKV has been detected in multiple organs of infected people, including immune-privileged sites like the brain, eyes, and reproductive tract. ZIKV replication in the reproductive tract is of high concern; ZIKV can be transmitted sexually or to the developing fetus of pregnant women, resulting in severe congenital defects. Here, we show that ZIKV-infected immunocompetent mice rapidly controlled viremia with no viral rebound following T cell depletion. In contrast, mice genetically deficient in B and T cells (immunodeficient mice) supported sustained ZIKV replication in all tissues examined. Treatment of ZIKV-infected immunodeficient mice with the novel nucleoside analog 7-deaza-7-fluoro-2′-C-methyladenosine (DFMA) significantly reduced viremia and prolonged survival, validating immunodeficient mice for efficacy studies of ZIKV prevention and therapeutic approaches. Importantly, we demonstrate that treatment of ZIKV-infected animals with a dengue virus cross-neutralizing antibody (EDE1, C10) suppressed systemic ZIKV replication, including in the brain, eye, and male and female reproductive tracts.

**IMPORTANCE:**

Since 2007, Zika virus (ZIKV) infections have been documented in over 80 countries and territories, resulting in two major outbreaks thus far. ZIKV has been detected in multiple organs of infected people, including immune-privileged sites like the brain, eyes, and reproductive tract. ZIKV replication in the reproductive tract is of high concern as ZIKV can be transmitted sexually or to the developing fetus of pregnant women, resulting in severe congenital defects. Currently, no effective therapy or vaccine exists to protect against the next outbreak. Here, we developed a preclinical animal model for ZIKV infection that we used to evaluate the efficacy of a dengue virus cross-neutralizing antibody for prevention/treatment of ZIKV infection. The antibody suppressed virus replication in blood and tissues, including the reproductive tract, suggesting that passive administration of ZIKV neutralizing antibodies could be used during future ZIKV outbreaks in high-risk populations to prevent ZIKV transmission.

## INTRODUCTION

Since the re-emergence of the mosquito-borne Zika virus (ZIKV) in 2007 (Yap Islands), ZIKV has persisted with major outbreaks occurring in 2013 (French Polynesia) and 2015 (Americas) (1–5). To date, ZIKV infections have been documented in over 80 countries and territories (6). Of concern, in addition to mosquito transmission, ZIKV can be transmitted vertically (mother-to-fetus) and horizontally (between male and female), a feature that sets ZIKV apart from other flaviviruses (7–9). Multiple tissues in the body can harbor ZIKV infection, including immune-privileged sites like the brain, eye, and gonads, which can lead to neurological disorders, conjunctivitis, retinal damage, testicular damage, infertility, and teratogenicity (10–15). ZIKV-RNA can be detected in the semen of men for over a year after the onset of symptoms, underscoring a potentially prolonged period in which men could sexually transmit ZIKV to their partners (16). Taken together, these data suggest an imminent need for a therapeutic intervention that can significantly reduce systemic ZIKV infection, especially from potential sites of virus transmission like the reproductive tract.

The inoculation of immunocompetent, wild-type mice (C57BL/6, BALB/c, or CD-1 mice) with ZIKV does not result in disease, and little to no infectious virus or viral RNA is detected in tissues. The resistance of immunocompetent mouse strains to ZIKV infection is due to the inability of ZIKV to antagonize the mouse type I interferon (IFN) response. As a result, most mouse models of ZIKV block the type I interferon response by using mice with genetic deficiencies in the type I IFN signaling pathway or by injecting mice with anti-IFNAR1 antibodies prior to virus exposure (17–21). ZIKV infection of immunodeficient mice, genetically deficient in T cells, B cells, and NK cells, has also been demonstrated (22, 23). Although critical parameters of infection, including viral replication kinetics, dissemination of virus to different tissues, and survival, have yet to be determined.

In this study, we show that ZIKV-infected immunocompetent BALB/c mice rapidly controlled viremia with no viral rebound following T cell depletion. Of interest, almost 1 year post-infection, ZIKV-RNA was detected in the eye of 2/8 mice analyzed, but in no other tissues examined. In contrast, two immunodeficient mouse strains, NOD/SCID and NSG, support high and sustained systemic levels of ZIKV replication. These mice are deficient in B cells and T cells (NOD/SCID) or B cells, T cells, and NK cells (NSG) but have a full complement of IFN and IFN-receptor genes. ZIKV infection resulted in high viremia, delayed signs of illness, and extended survival. Importantly, ZIKV disseminated to all tissues tested, including highly clinically relevant tissues like the brain, eyes, gastrointestinal tract, and both male and female reproductive organs. We then showed that treatment with the novel nucleoside analog 7-deaza-7-fluoro-2′-C-methyladenosine (DFMA) significantly reduced viremia in ZIKV-infected immunodeficient mice and prolonged survival, validating immunodeficient mice as a model to evaluate the efficacy of ZIKV prevention and therapeutic approaches. Subsequently, we used this prolonged ZIKV infection model to evaluate the *in vivo* efficacy of a dengue virus monoclonal antibody (C10) with cross-neutralizing potential that binds the envelope-dimer epitope 1 (EDE1) for prevention of ZIKV infection. A single dose of C10 antibody was able to provide efficient and sustained suppression of viremia and a significant reduction in ZIKV-RNA levels in all tissues analyzed, including the male and female reproductive tracts, demonstrating the strong therapeutic potential for monoclonal antibody therapy during future ZIKV outbreaks.

## RESULTS

### Immunocompetent mice effectively control ZIKV infection

**Fig 1:**
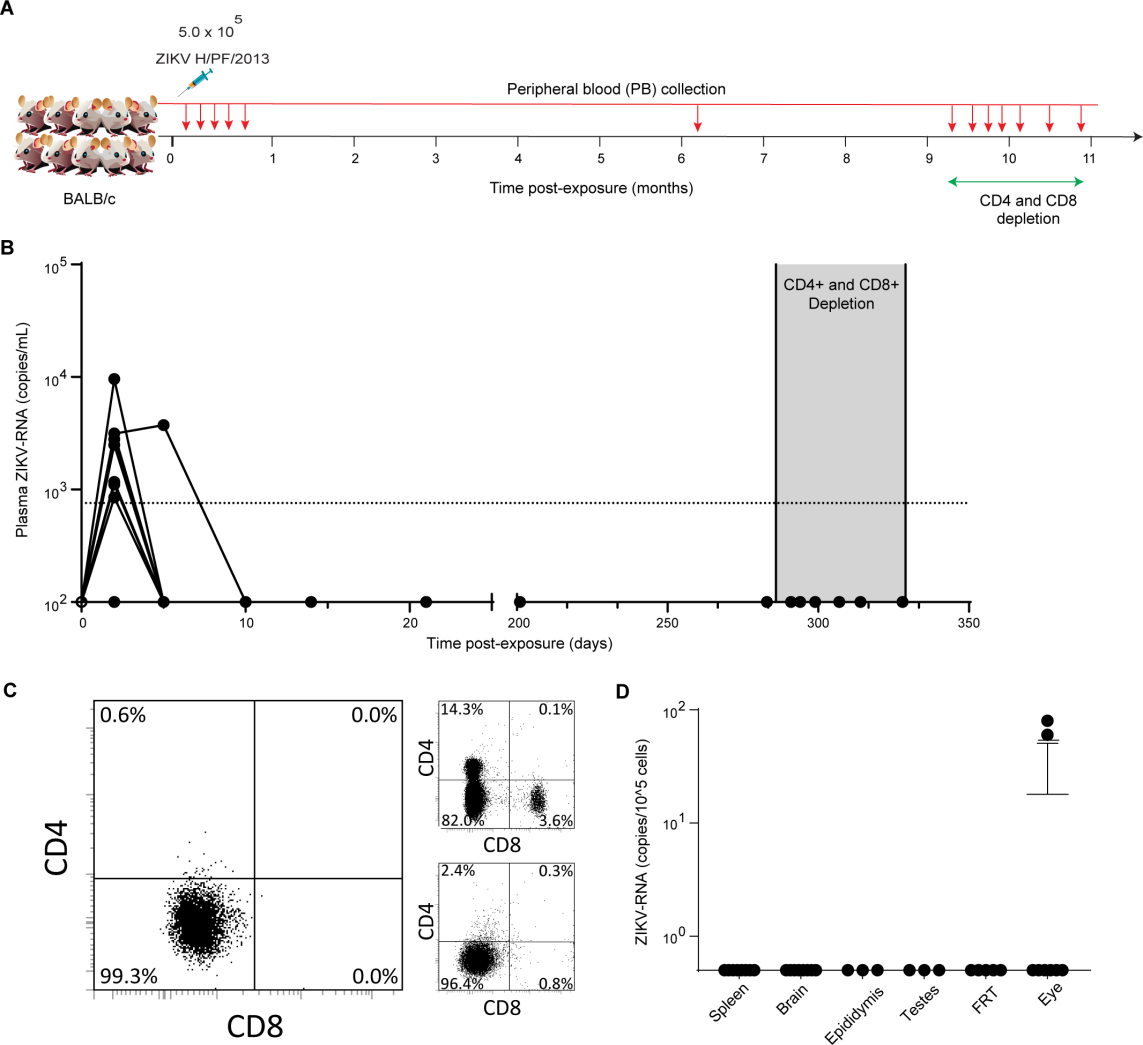
BALB/c mice effectively control ZIKV infection, but virus can persist in the eye. (**A**) Experimental design. BALB/c mice (*n* = 10) were intravenously exposed to ZIKV H/PF/2013 (5.0 × 10^5^ FFU) and monitored over time for the presence of ZIKV-RNA in peripheral blood plasma (small red arrows). Mice were depleted of CD4^+^ and CD8^+^ T cells at 286 days post-exposure using anti-mCD4^+^ (GK1.5) and anti-mCD8^+^ (2.43) antibody treatment. Cell-associated ZIKV-RNA levels in multiple tissues were analyzed after 42 days of T-cell depletion. (**B**) ZIKV-RNA levels in the plasma of ZIKV-exposed BALB/c mice (*n* = 10). The shaded area represents the period of antibody treatment. Dashed line: ZIKV-RNA limit of detection (833 copies/mL). (**C**) Flow cytometric analysis of peripheral blood confirming CD4^+^ and CD8^+^ T cell depletion in ZIKV-infected BALB/c mice after treatment with anti-T cell depleting antibodies (left). Flow cytometric analysis of the spleen at the time of harvest showed the presence of T cells in untreated mice (top right) and the depletion of T cells in treated mice (bottom right). Flow cytometry gating strategy: singlets→live cells→mCD45^+^→mCD3^+^→mCD4^+^ or mCD8^+^. (**D**) ZIKV-RNA levels in the tissues of BALB/c mice harvested at 328 days post-exposure and 42 days after CD4^+^ and CD8^+^ T cell depletion (spleen, brain, eye, *n* = 8; epididymis, testes, *n* = 3; female reproductive tract [FRT], *n* = 5). Horizontal and vertical lines represent the mean and standard deviation, respectively.

Immunocompetent mice rapidly clear ZIKV from the periphery without any visible symptoms (19, 24). However, ZIKV persistence after post-acute phase clearance from peripheral blood in such mice remains unexplored. To evaluate ZIKV persistence in immunocompetent mice, 10 BALB/c mice were intravenously infected with the ZIKV H/PF/2013 strain (Fig. 1A). Following ZIKV exposure, ZIKV-RNA levels were measured at least once weekly in the peripheral blood plasma of mice for 28 days. Two days after exposure, ZIKV-RNA was detected in the plasma of 9/10 animals (mean = 3.4 × 10^3^ ZIKV-RNA copies/mL) (Fig. 1B). By 10 days post-exposure, ZIKV-RNA was undetectable in the plasma of all animals, and no viral blips or rebounds were subsequently detected in any animal through day 28 post-exposure. Plasma ZIKV-RNA levels were analyzed again at 6 months (201 days) and 9 months (283 days) post-exposure, which confirmed long-term suppression of viremia.

Recently, a study demonstrated that immunocompetent neonatal mice exposed to ZIKV exhibited viral rebound upon immunosuppression in adulthood (25). To investigate the possibility of adaptive immune cell-mediated control of viral replication, CD4^+^ T cells and CD8^+^ T cells were depleted from these animals for 42 days (Fig. 1A). CD4^+^ and CD8^+^ T cell depletion was confirmed in peripheral blood by flow cytometry (Fig. 1C, left). Despite the effective depletion of both CD4^+^ and CD8^+^ T cells, no viral rebound was detected in peripheral blood. Finally, at 42 days after T cell depletion (328 days post-ZIKV exposure), mice were necropsied for further analysis. Flow cytometric analysis demonstrated efficient CD4^+^ and CD8^+^ T cell depletion in tissues (Fig. 1C, right bottom). No ZIKV-RNA was detected in any of the samples analyzed from the spleen, brain, male reproductive tract (MRT, i.e., epididymis, testes), or female reproductive tract (FRT) (Fig. 1D). However, several hundred copies of ZIKV-RNA were detected in the eyes of 2/8 mice analyzed (Fig. 1D). These results demonstrate that despite efficient control and clearance of ZIKV from the peripheral blood and tissues, in some animals, ZIKV-RNA can persist in the eyes for almost a year post-exposure.

### Immunodeficient mice are permissive to ZIKV infection and sustain high levels of ZIKV replication

**Fig 2:**
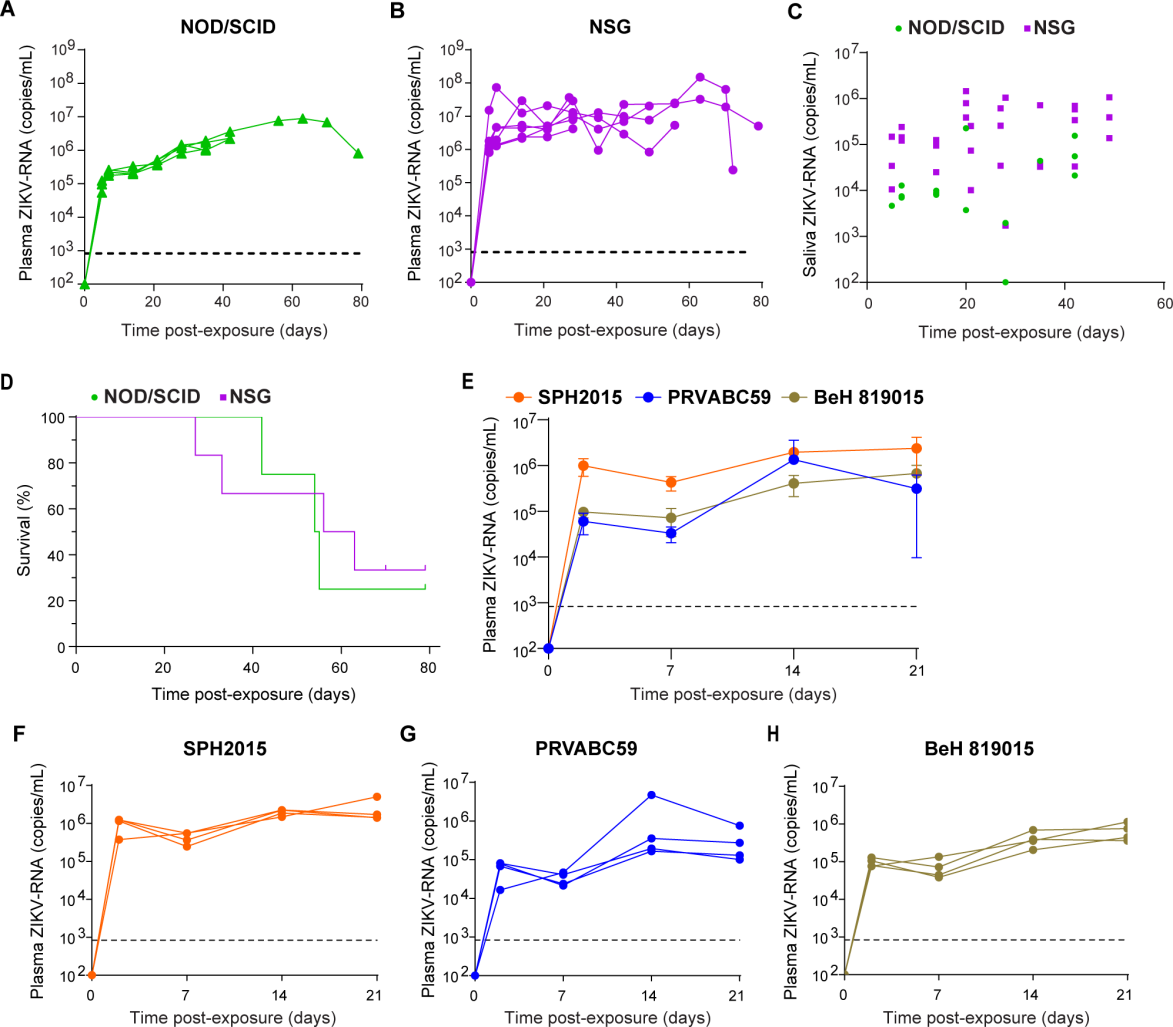
Sustained viremia and viral shedding in the saliva of ZIKV-infected immunodeficient mice. ZIKV-RNA levels in the plasma of (**A**) NOD/SCID (*n* = 4) and (**B**) NSG (*n* = 6) immunodeficient mice intravenously exposed to ZIKV H/PF/2013 (0.5–1.0 × 10^6^ FFU). (**C**) ZIKV-RNA levels in saliva of NOD/SCID (*n* = 4, green circles), and NSG (*n* = 6, purple squares) mice. (**D**) Kaplan-Meier plot comparing the survival of ZIKV-infected NOD/SCID (*n* = 4, green line) and NSG (*n* = 6, purple line) mice. (**E–H**) Plasma ZIKV-RNA levels in NSG mice intravenously exposed to 5.0 × 10^5^ FFU ZIKV strains PRVABC59 (*n* = 4), SPH2015 (*n* = 4), or BeH 819015 (*n* = 4) are shown as (**E**) a group or (**F–H**) individual mice. Horizontal and vertical lines represent the mean and standard deviation, respectively. Dashed line: ZIKV-RNA limit of detection (833 copies/mL).

We exposed two immunodeficient mouse strains, NOD/SCID (deficient in T cells and B cells) and NSG (deficient in T cells, B cells, and NK cells), to ZIKV H/PF/2013. Approximately 1,000-fold higher levels of ZIKV-RNA in plasma were detected at 2 days post-exposure in NOD/SCID and NSG mice compared to BALB/c mice (Fig. 2A and B). Contrary to BALB/c mice, longitudinal analysis demonstrated high and sustained levels of ZIKV-RNA in the plasma of both strains of immunodeficient mice (Fig. 2A and B). Similar plasma ZIKV-RNA levels were observed in male and female NSG mice, indicating no notable gender bias in ZIKV replication levels (Fig. S1A). We also verified that ZIKV in the peripheral blood of infected NSG mice is replication competent and capable of establishing a new infection using an *in vitro* VERO cell-based infection assay and by injecting naïve NSG mice with serum collected from ZIKV-infected NSG mice at days 21 and 28 post-infection (Fig. S2).

The presence and persistence of ZIKV were evaluated in the saliva, urine, and cervicovaginal secretions (CVS) of mice exposed to ZIKV. High and sustained levels of ZIKV-RNA were detected in the saliva of ZIKV-infected immunodeficient mice (Fig. 2C). Similarly, ZIKV-RNA was also detected in the urine and CVS samples collected from ZIKV-infected mice longitudinally (Table S1). We further observed that, contrary to BALB/c mice, which survived for almost a year post-ZIKV infection (Fig. 1B), immunodeficient mice succumbed to infection over time. Half of ZIKV-infected NOD/SCID and NSG mice succumbed to infection by 54 and 56 days post-exposure, respectively (Fig. 2D). No significant differences in survival (*P* = 0.8317) were observed between male and female ZIKV-infected mice (Fig. S1B).

To establish the ability of different strains of ZIKV to replicate in immunodeficient mice, we inoculated NSG mice with three additional strains of ZIKV from prior outbreaks in the western hemisphere, including one strain from Puerto Rico (PRVABC59) and two strains representing distinct ZIKV clades from Brazil (SPH2015 and BEH 819015). Within 2 days of exposure, infection with each strain resulted in robust levels of ZIKV-RNA in the plasma of mice, which were sustained over time (Fig. 2E through H). Taken together, these results demonstrate the susceptibility of immunodeficient mouse strains to ZIKV infection, characterized by robust and sustained viremia and extended survival compared to some other commonly used mouse models (17–19, 24, 26, 27), without the need for the administration of pathogenesis-enhancing interventions like anti-IFN treatment.

### Systemic replication of ZIKV in tissues of immunodeficient mice

**Fig 3:**
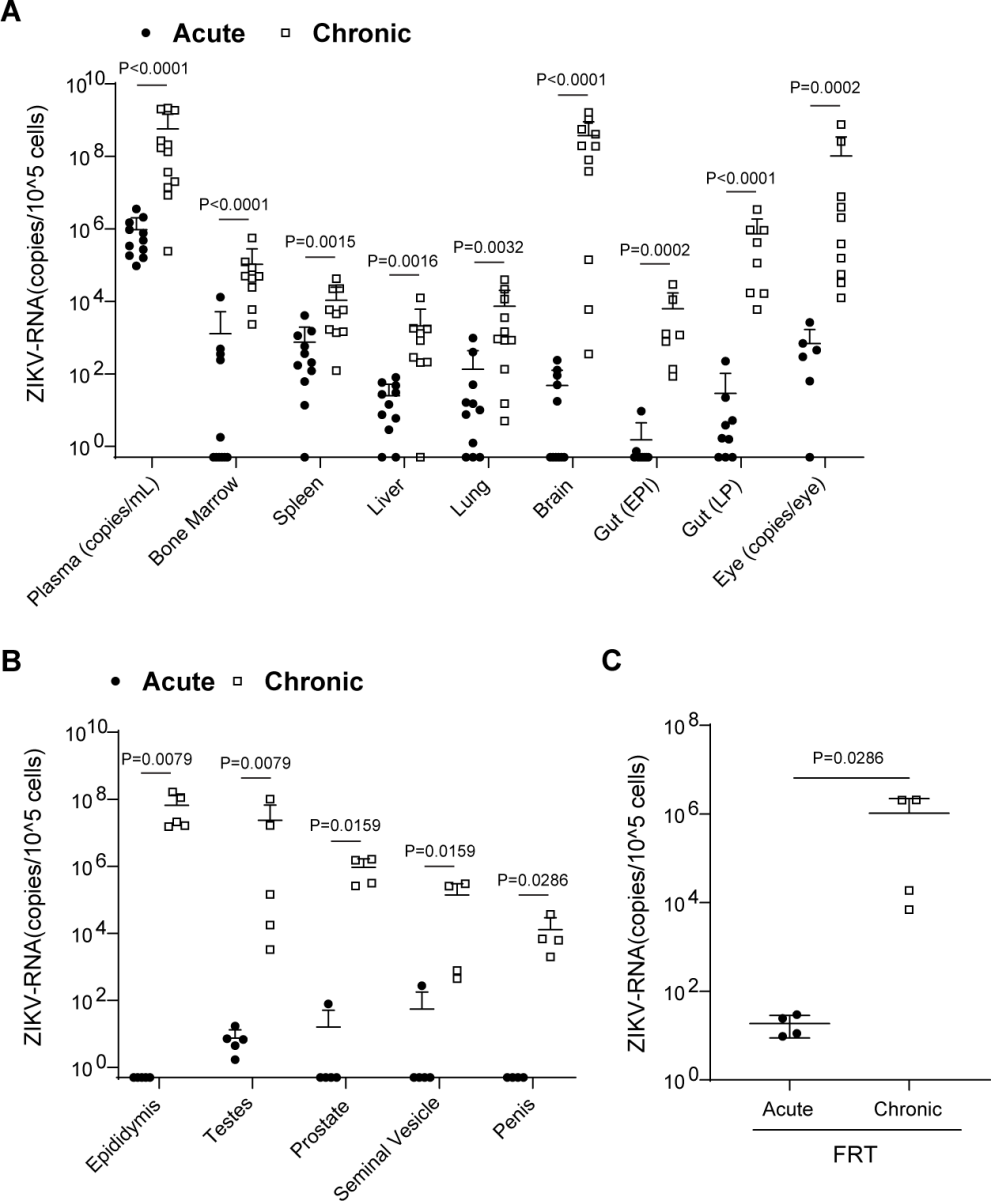
Analysis of systemic infection in immunodeficient mice exposed to ZIKV. Mice were intravenously exposed to ZIKV H/PF/2013 (0.25–1 × 10^6^ FFU) and analyzed during acute (2 days post-infection, *n* = 11) or chronic (27 to 73 days post-infection, *n* = 12) infection. (**A**) ZIKV-RNA levels in plasma (acute, *n* = 11; chronic, *n* = 12) and cells isolated from the bone marrow (acute, *n* = 11; chronic, *n* = 9), spleen (acute, *n* = 11; chronic, *n* = 10), liver (acute, *n* = 11; chronic, *n* = 9), lung (acute, *n* = 11; chronic, *n* = 11), brain (acute, *n* = 11; chronic, *n* = 11), gut epithelium (gut EPI; acute, *n* = 9; chronic, *n* = 7), gut lamina propria (gut LP; acute, *n* = 9; chronic, *n* = 8), and eyes (acute, *n* = 6; chronic, *n* = 10) of acutely or chronically infected mice. For the plasma and the eye, ZIKV-RNA levels were calculated per milliliter and one whole eye, respectively. ZIKV-RNA levels in cells isolated from the (**B**) male reproductive tract; epididymis (acute, *n* = 5; chronic, *n* = 5), testes (acute, *n* = 5; chronic, *n* = 5), prostate (acute, *n* = 5; chronic, *n* = 4), seminal vesicles (acute, *n* = 5; chronic, *n* = 4), penis (acute, *n* = 4; chronic, *n* = 4), and (**C**) female reproductive tract (FRT; acute, *n* = 4; chronic, *n* = 4) of acute and chronically infected mice. Horizontal and vertical lines represent the mean and standard deviation, respectively. For the statistical analysis of ZIKV-RNA levels, a Mann-Whitney test was performed.

To establish systemic replication of ZIKV during acute and chronic phases of infection, ZIKV-RNA levels were evaluated in the peripheral blood plasma and cells isolated from the bone marrow, spleen, liver, lung, brain, gut epithelium, gut lamina propria, and eyes of acute (2 days post-exposure) and chronically (27–73 days post-exposure) infected NSG mice. During acute ZIKV infection (2 days post-exposure), high levels of ZIKV-RNA were detected in the plasma of all animals; however, the levels of cell-associated ZIKV-RNA were variable in the different tissues analyzed (Fig. 3A). While ZIKV-RNA was readily detected in the spleen, liver, lung, gut lamina propria, and eyes of most mice, ZIKV-RNA was detected in less than half of the bone marrow, gut epithelium, and brain samples analyzed (Fig. 3A). In contrast, during chronic ZIKV infection, significantly higher ZIKV-RNA levels were detected in all tissues analyzed (Fig. 3A). Of these tissues, the highest levels of ZIKV-RNA were present in the brain and the eye (Fig. 3A). These results demonstrate that ZIKV rapidly establishes a systemic infection in immunodeficient mice that is maintained at very high levels during chronic infection in all tissues analyzed.

### ZIKV replicates in the male and female reproductive tract of immunodeficient mice

Given the risk for sexual transmission of ZIKV, we also determined the presence of ZIKV in the MRT (testes, epididymis, prostate, penis, and seminal vesicles) and FRT of ZIKV-infected NSG mice. Remarkably, at 2 days post-infection, detectable levels of ZIKV-RNA were present in the testes of all male mice and in the FRT of all female mice (Fig. 3B and C). Although we did not consistently observe the presence of ZIKV-RNA in MRT tissues during acute infection (Fig. 3B), ZIKV-RNA was consistently detected and at significantly higher levels in all tissues (i.e., epididymis, testes, prostate, seminal vesicle, and penis) of the MRT during chronic infection. Interestingly, the epididymis and testes had the highest viral burden in the MRT, followed by the prostate, seminal vesicles, and the penis (Fig. 3B). Similarly, the levels of ZIKV-RNA in the FRT were significantly higher during chronic infection (Fig. 3C). These results demonstrate that ZIKV is present in the male and female reproductive tracts during acute ZIKV infection and that ZIKV replication is sustained at high levels in these compartments during chronic infection.

### Antiviral treatment reduces viremia and improves survival after ZIKV infection

**Fig 4:**
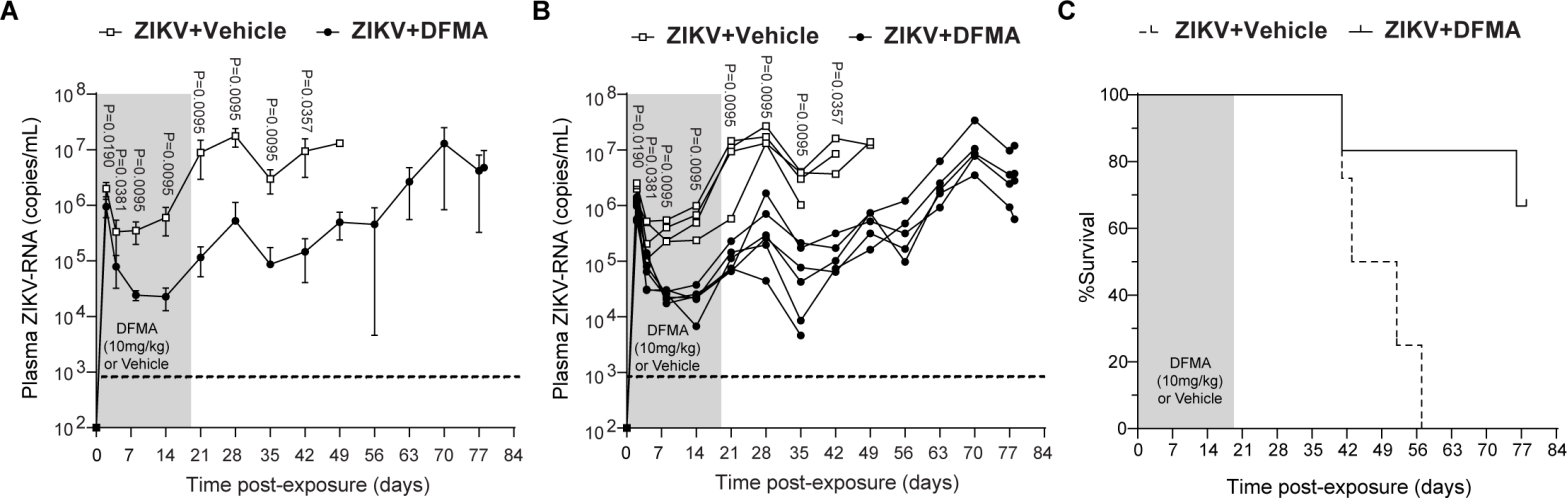
Treatment of ZIKV-infected mice with DFMA reduces viremia and improves survival. (**A–C**) NSG mice infected with ZIKV H/PF/2013 (2.5 × 10^5^ FFU) received daily DFMA (10 mg/kg, *n* = 6 mice) or vehicle (control, *n* = 4 mice) treatment starting 2 days prior to exposure. Drug administration was continued for a total of 21 days (in gray). Plasma ZIKV-RNA levels of DFMA (black circles) and vehicle (white squares) treated mice in (**A**) aggregate or (**B**) individually. ZIKV-RNA was quantified by reverse transcription-PCR (RT-PCR), and the limit of detection (833 copies per milliliter) is noted with a dashed line. ZIKV-RNA levels between DFMA-treated and control mice were compared up to 42 days post-exposure using a Mann-Whitney test. (**C**) Kaplan-Meier plot illustrating the post-exposure survival of DFMA-treated (solid line) and vehicle control (dashed line) mice (*P* = 0.0179), Mantel-Cox log-rank test.

Currently, there are no approved treatments for ZIKV infection. The nucleoside analog DFMA reduces ZIKV replication *in vitro* and *in vivo* in IFN-deficient mice (28). To assess the utility of immunodeficient mice to perform efficacy studies of ZIKV prevention and therapeutic approaches, we evaluated DFMA therapy in NSG mice. Beginning 2 days prior to infection with ZIKV H/PF/2013, NSG mice were administered DFMA (10 mg/kg per day) or vehicle daily for 21 days (until 19 days post-exposure). Plasma ZIKV-RNA levels were monitored over time. Four days after an intravenous ZIKV exposure, ZIKV-RNA levels in the plasma of DFMA-treated mice were 75% lower (*P* = 0.0381) compared to vehicle-treated controls, and by 8 days post-ZIKV exposure, ZIKV-RNA levels were >90% lower (*P* = 0.0095) in DFMA-treated animals (Fig. 4A and B). After the discontinuation of DFMA treatment, plasma ZIKV-RNA levels increased over time (Fig. 4A and B). However, compared to vehicle-treated mice, significantly lower levels of viremia were observed in mice that had received DFMA treatment for up to 3 weeks post-treatment discontinuation (Fig. 4A and B). DFMA treatment also prolonged the survival of ZIKV-infected mice, significantly reducing mortality (Fig. 4C, *P* = 0.0179). These results demonstrate that DFMA treatment reduces viremia and delays mortality during infection and validates immunodeficient mice as a model to evaluate the efficacy of ZIKV prevention and therapeutic approaches.

### Pretreatment with C10, a ZIKV-neutralizing antibody, markedly reduces viremia

**Fig 5:**
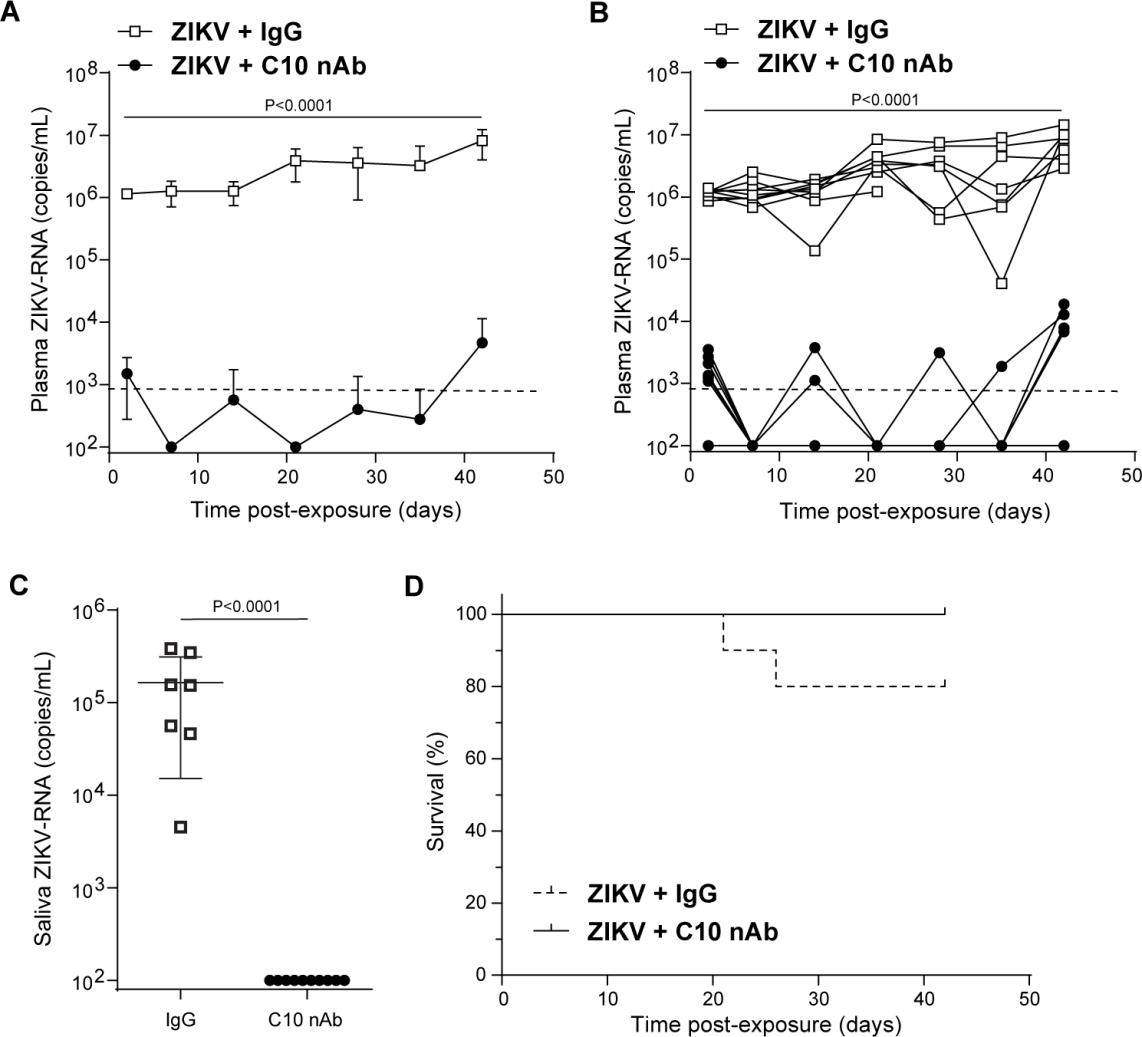
C10-neutralizing antibody administration dramatically reduces ZIKV replication and prevents viral shedding. ZIKV-RNA levels in the plasma of mice administered a single dose of C10 monoclonal antibody (62.5 µg; dark circles, *n* = 10 mice) or IgG control antibody (white boxes, *n* = 9 mice) 18 h before intravenous exposure to ZIKV H/PF/2013 (2.5 × 10^5^ FFU) shown for the (**A**) group or (**B**) individual mice. Dashed line: ZIKV-RNA limit of detection (833 copies/mL). (**C**) ZIKV-RNA levels in saliva collected from C10-treated (*n* = 10; dark circles) and control mice (*n* = 7; white boxes) at 30 days post-ZIKV exposure. (**D**) Kaplan-Meier plot illustrating the survival of C10-treated and control mice. ZIKV-RNA levels between C10-treated and control mice were compared using a Mann-Whitney test. Horizontal and vertical lines represent the mean and standard deviation, respectively.

C10 is a dengue virus serotype cross-neutralizing monoclonal antibody that was isolated from a dengue patient (29–31). C10 antibody treatment prevented weight loss and increased survival in ZIKV-infected type I/II interferon receptor-knockout mice (30). However, its effect on systemic Zika viral load levels was not determined. Therefore, we performed a comprehensive preclinical investigation of the effect of C10 pre-exposure prophylaxis on the establishment of ZIKV infection and replication. NSG mice received one systemic dose of C10 antibody or IgG control antibody. Mice were then exposed intravenously to ZIKV H/PF/2013 18 h after antibody treatment, and ZIKV infection was monitored in peripheral blood plasma for 6 weeks. All mice treated with control antibody acquired ZIKV infection. High levels of ZIKV-RNA were detected in the plasma of all control animals within 2 days of exposure and were maintained for 6 weeks (last time point analyzed) (Fig. 5A and B). On the contrary, there was no evidence of infection in the peripheral blood of 3/10 mice that received one dose of C10 antibody at 2 days post-exposure. Low levels of ZIKV-RNA (mean = 2.10 × 10^3^, SD = 9.20 × 10^2^ copies/mL) were only transiently observed in plasma at 2 days post-exposure in 7/10 animals (Fig. 5B). By day 7 post-infection, plasma ZIKV-RNA levels were below the limit of detection in all C10-treated mice (Fig. 5B). ZIKV-RNA was undetectable in plasma through 35 days post-exposure, with only four transient blips above the limit of detection in 3/10 C10-treated animals. At 6 weeks post-exposure, the levels of ZIKV-RNA remained undetectable in 6/10 C10-treated mice. ZIKV-RNA was detected in the plasma of 4/10 C10-treated mice (mean = 1.15 × 10^4^, SD = 5.53 × 10^3^ copies/mL), but the overall levels were 3 logs lower (*P* = 0.0061) compared to control antibody-treated mice. These results indicate that while a single dose of C10 antibody was not able to prevent ZIKV infection following an intravenous challenge, antibody treatment significantly suppressed viremia for 6 weeks with only transient blips of viremia observed.

### C10 antibody treatment significantly inhibited ZIKV-RNA shedding

Interestingly, ZIKV-RNA was not detected in saliva collected from any (0/10) C10-treated mouse at 30 days post-ZIKV infection. In contrast, high levels of ZIKV-RNA were present in the saliva of control antibody-treated mice (mean = 1.64 × 10^5^, SD = 1.48 × 10^5^ copies/mL) (Fig. 5C). By 26 days post-exposure, 2/9 control antibody-treated mice succumbed to infection, consistent with half of ZIKV-infected NSG mice. Notably, no C10 antibody-treated mice succumbed to infection during the experiment (Fig. 5D). These data demonstrate that a single dose of C10 antibody administered before ZIKV exposure effectively inhibits ZIKV replication *in vivo* over an extended period and markedly reduces ZIKV-RNA levels in plasma (*P* < 0.0001) and saliva (*P* < 0.0001).

### A single dose of C10 antibody significantly reduces ZIKV replication in tissues

**Fig 6:**
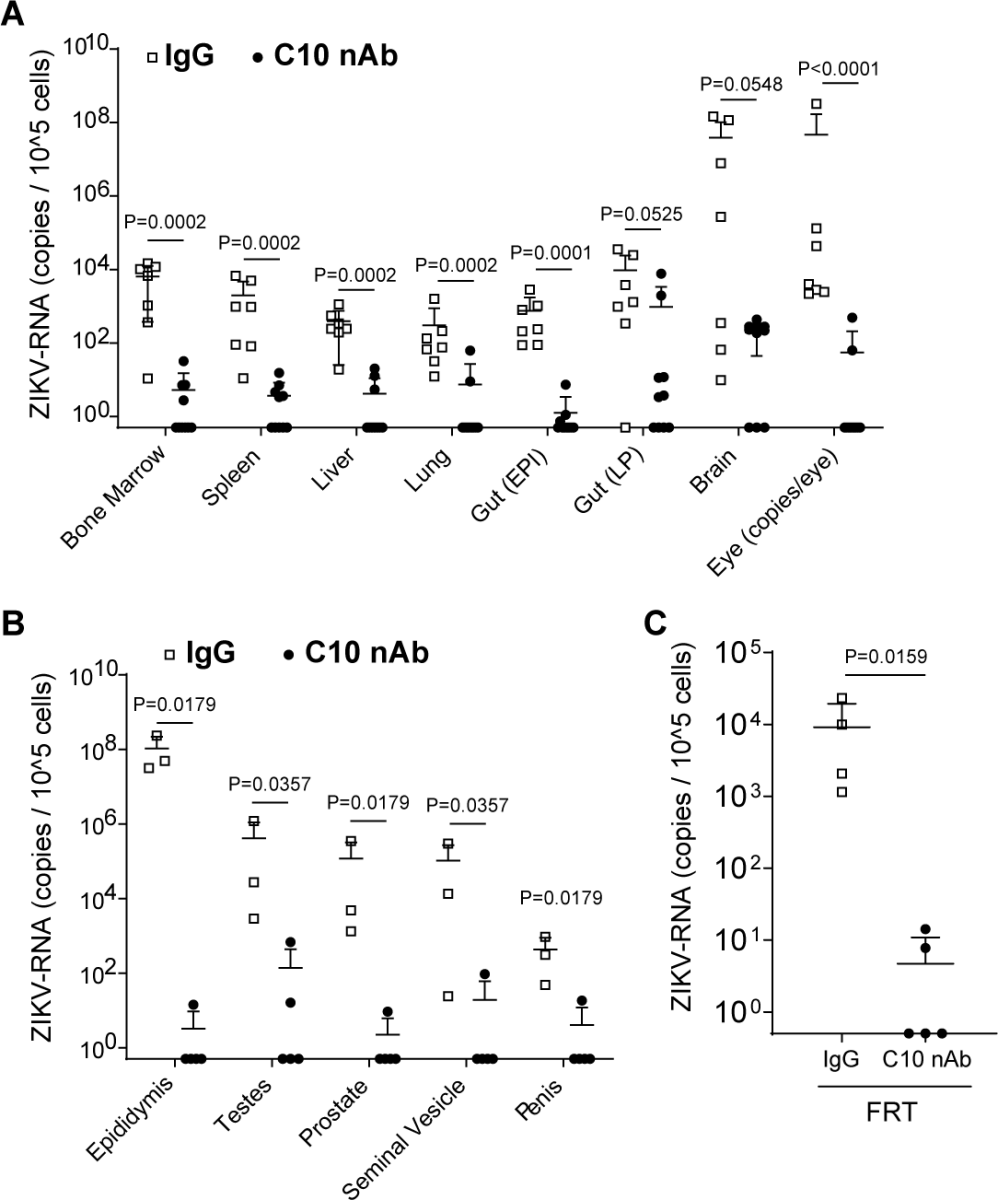
C10 antibody administration effectively reduces ZIKV replication in tissues. Mice administered C10 monoclonal antibody (*n* = 10; dark circles) or IgG control antibody (*n* = 9; white boxes) 18 h before intravenous exposure to ZIKV H/PF/2013 (2.5 × 10^5^ FFU). Mice were necropsied at 42–44 days post-exposure to analyze ZIKV-RNA levels in tissues. ZIKV-RNA levels in cells isolated from the (**A**) bone marrow (BM; C10, *n* = 10; IgG, *n* = 7), spleen (C10, *n* = 10; IgG, *n* = 7), liver (C10, *n* = 10; IgG, *n* = 7), lung (C10, *n* = 10; IgG, *n* = 7), gut epithelium (gut EP; C10, *n* = 10; IgG, *n* = 7), gut lamina propria (gut LP; C10, *n* = 10; IgG, *n* = 7), eye (C10, *n* = 10; IgG, *n* = 7), and the (**B**) male reproductive tract (epididymis, penis, prostate, seminal vesicles, testes; C10, *n* = 5; IgG, *n* = 3) and (**C**) female reproductive tract (FRT; C10, *n* = 5; IgG, *n* = 4). ZIKV-RNA levels between C10-treated and control mice were compared using a Mann-Whitney test. Horizontal and vertical lines represent the mean and standard deviation, respectively.

At 6 weeks post-exposure, ZIKV-RNA levels were evaluated in tissues from ZIKV-infected mice treated with C10 or control antibody. Cells were isolated and analyzed from the bone marrow, spleen, liver, lung, gut epithelium, gut lamina propria, brain, and eyes (Fig. 6A). In control antibody-treated mice, ZIKV-RNA was readily detected in all tissues analyzed from all animals except for a gut lamina propria sample from one mouse. The highest levels of ZIKV-RNA were observed in the gut lamina propria, brain, and eyes. Conversely, almost all tissues analyzed from C10 antibody-treated mice had either undetectable or low levels of viral RNA. In most tissues of C10 antibody-treated mice, ZIKV-RNA levels were 2–5 logs lower compared to control animals (Fig. 6A). These results underscore the effectiveness of the C10 antibody in penetrating and significantly inhibiting viral replication in tissues after a single administration (Fig. 6A).

### C10 antibody efficiently suppresses ZIKV replication in the male and female reproductive tracts

Because of their relevance to sexual ZIKV transmission, we evaluated the effect of C10 pretreatment on the levels of the virus in the different organs of the MRT (testes, epididymis, prostate, penis, and seminal vesicles) and FRT of ZIKV-infected mice. ZIKV-RNA was readily detected in the epididymis, penis, prostate, seminal vesicles, and testes of all control antibody-treated male animals (Fig. 6B). In contrast, ZIKV-RNA levels were significantly lower in all the male genital tract tissues from ZIKV-infected-C10-treated animals, and in most samples, ZIKV-RNA was not detected (Fig. 6B). ZIKV-RNA was also detected in the FRT of all female animals administered the control antibody. However, significantly lower levels of ZIKV-RNA were observed in the FRT of C10 antibody-treated animals (Fig. 6C). Collectively, these results demonstrate that the C10 antibody efficiently penetrates the male and female reproductive tracts and that a single dose of antibody significantly inhibits ZIKV replication in these tissues for at least 6 weeks.

## DISCUSSION

Our results demonstrate that mouse strains deficient in T, B, and NK cells support sustained systemic ZIKV replication, particularly in highly clinically relevant tissues like the brain, eyes, gastrointestinal tract, and both male and female reproductive tracts. ZIKV-infected immunodeficient mice also exhibited extended survival compared to some other commonly used mouse models (17–19, 24, 26, 27), allowing for the evaluation of preventative and therapeutic approaches.

During human ZIKV infection, the viral NS5 protein inhibits STAT2, thereby suppressing the type I IFN response to ZIKV, allowing for viral replication and dissemination (19, 24, 32). However, in immunocompetent mice, NS5 cannot effectively bind mouse STAT2, resulting in a robust IFN response that suppresses ZIKV replication and infection (32, 33). This is consistent with our results showing that although BALB/c mice were permissive to ZIKV H/PF/2013 infection, plasma ZIKV-RNA levels quickly decreased below the level of detection in all animals by 10 days post-infection. Depletion of mouse T cells after viremia was suppressed did not result in rebound of viremia. These results suggest that BALB/c mice cleared infection and/or that rebound was prevented by an innate and/or ZIKV antibody response. Of note, two immunocompetent mice were positive for ZIKV in the eyes even after 300 days of infection. In humans, the presence of ZIKV has been shown in tears 30 days post-illness (34). In this study, only 3/29 patients had detectable virus in the eye. Studies in humans have also demonstrated that ZIKV infects the cornea, iris, optic nerve, ganglion, and bipolar cells in the retina and that infection can persist in the eye, resulting in retinitis, focal retinal degeneration, and ganglion cell loss (13, 14, 20).

Growing evidence suggests that the immune response to ZIKV infection in mice is more complex than just the type I IFN response and that other components of the immune system contribute to the control of ZIKV infection. For example, wild-type C57BL/6 mice treated with anti-IFNAR antibodies have a suppressed, but not completely deficient, IFN response and develop viremia when exposed to Asian ZIKV strains. However, they do not lose weight or develop neurologic disease (24). Mice deficient in mitochondrial antiviral signaling, an adaptor of cytosolic RIG-I-like receptors signaling, develop an acute infection after ZIKV exposure but only experience significant weight loss when their T cells are depleted (35). ZIKV-infected AIR mice, Rag1^−/−^ mice deficient in functional T and B cells and treated with anti-IFNAR antibody, have high levels of ZIKV-RNA in the spleen, lymph nodes, and brain and exhibit significant weight loss (35). Thus, both adaptive and innate immune responses are involved in the control of ZIKV replication, both its spread and the severity of disease symptoms.

NOD/SCID and NSG immunodeficient mice are deficient in T cells, B cells, and C5 complement and have reduced myeloid and dendritic cell activity. NK cell activity is also impaired in NOD/SCID mice, while NSG mice are deficient in NK cells due to a common gamma chain null mutation (γc^−/−^). These mice have a full complement of IFN and IFN-receptor genes; however, it is possible that the IFN levels are reduced due to lower myeloid and dendritic cell activity. Our data showed that immunodeficient mice infected with ZIKV had robust plasma viremia detected as early as 2 days post-infection that was sustained for up to 79 days. Although lower plasma viral loads were observed in NOD/SCID mice at early time points post-exposure compared to NSG mice, ZIKV-RNA levels increased over time in the plasma of NOD/SCID mice. Sustained plasma viremia has also been observed in ZIKV-infected humanized and non-humanized Rag2^−/−^γc^−/−^ immunodeficient mice (22, 36). ZIKV-RNA was detected in multiple tissues as early as 2 days post-infection, suggesting rapid tissue dissemination. Given the prolonged infection and high viral loads observed, an investigation of ZIKV genomic evolution over time may reveal whether ZIKV adaptation could contribute to the observed increase in viral loads during chronic versus acute phases. The survival of ZIKV-infected mice in NOD/SCID and NSG was much longer than some other murine models of ZIKV infection, where most of the animals died within 30 days or less (17–19, 24, 26, 27). Survival timelines in ZIKV mouse models are influenced by dose and route of exposure. A shorter survival timeline was observed in NOD/SCID mice exposed to a high-dose ZIKV challenge (10^8^ PFU) (23). Additionally, immunodeficient mice had high levels of ZIKV-RNA detected in their bodily fluids like saliva and female cervicovaginal secretions. ZIKV-RNA was also detected in all tissues analyzed from ZIKV-infected immunodeficient mice, including the bone marrow, liver, spleen, lung, brain, gut, and eye. Interestingly, the brain had the highest levels of ZIKV-RNA. This is corroborated by studies demonstrating that ZIKV can infect brain cells by crossing the blood-brain barrier after infecting endothelial cells and disrupting tight junctions (37–39). In the future, it would be of interest to assess ZIKV levels in the heart of immunodeficient mice since ZIKV infection has been implicated in cardiac complications (40) and to evaluate infection with more virulent strains of ZIKV, including the African strain MR-766.

Recent studies suggest that the incidence of sexual transmission of ZIKV is underestimated and may contribute to the persistence of ZIKV in the human population (7, 41, 42). ZIKV-RNA has been detected in the vaginal secretions of women infected with ZIKV, which could transmit ZIKV to their sexual partners or, if pregnant, to the developing fetus (43, 44). Similarly, the presence of ZIKV-RNA has been reported in the semen of convalescent men even a year after symptom onset, underscoring a potentially long period of time in which men could sexually transmit ZIKV to a partner (11). In agreement with these studies, we observed ZIKV-RNA in different regions of the MRT (epididymis, testes, prostate, seminal vesicles, and penis) in ZIKV-infected immunodeficient mice months after virus exposure (15). Similarly, ZIKV-RNA was present in the cervicovaginal secretions and FRT of immunodeficient mice. Therefore, it will be necessary for successful ZIKV therapeutic interventions to suppress infection in the male and female reproductive tracts. Future studies will evaluate the potential for sexual or vertical transmission in immunodeficient mice. The fact that ZIKV was consistently detected in the saliva of infected animals is also noteworthy as it indicates the possibility of oral transmission. However, saliva collected from ZIKV-infected macaques was unable to transmit infection when applied directly to mucosal surfaces of naïve macaques, indicating that the relative risk of ZIKV transmission through saliva is likely low (45).

Currently, there are no antivirals or biologics specifically approved to prevent or treat ZIKV infection. At the dose and frequency tested, DFMA did not prevent or clear ZIKV infection following an intravenous exposure. Consistent with the results obtained in IFN knockout mice (28), DFMA treatment resulted in significant reductions in plasma viral loads that extended beyond the period of treatment and resulted in improved survival of infected animals. In this study, we did not measure ZIKV-RNA levels in the tissues of DFMA and vehicle-treated mice as we continued to monitor plasma ZIKV-RNA levels for several weeks after DFMA treatment ceased. However, based on the significantly lower plasma viral loads in mice during the DFMA treatment period compared to controls, we anticipate that during DFMA treatment, the levels of ZIKV-RNA in tissues would also be reduced. In the future, viruses in tissues could be sequenced to assess the development of drug resistance mutations. Reduced viral levels during the DFMA treatment period and for the 3 weeks following treatment cessation likely contributed to the increase in survival in DFMA-treated mice. Future studies will indicate the ability of DFMA treatment to reduce established ZIKV infection. Collectively, these results validate immunodeficient mice for future studies to test the efficacy of antivirals targeting ZIKV. We also evaluated the *in vivo* efficacy of a cross-reacting monoclonal antibody (C10) against EDE1. Previous studies demonstrated that another cross-reactive EDE1 antibody (EDE1-B10) significantly increased survival and reduced the viral burden in the serum, brain, eye, and male reproductive tissues of anti-Ifnar1 antibody-treated mice when administered within 3 days of ZIKV exposure (46). The C10 antibody was previously shown to neutralize ZIKV in cell culture and reduce ZIKV-induced morbidity and mortality in a type I/II IFN receptor-knockout murine model (31). In our study, C10 antibody treatment significantly reduced plasma viremia in immunodeficient mice immediately after virus exposure. A single dose of C10 antibody reduced ZIKV-RNA levels in plasma below or near the detection level for 6 weeks after infection. C10 antibody treatment also effectively reduced ZIKV-RNA shedding in saliva and ZIKV-RNA levels in all tissues examined, including the brain, eye, and male and female reproductive tracts, immune-privileged organs with the high levels of ZIKV replication. While multiple studies have documented C10 neutralization of Zika virus *in vitro*, it is possible that non-neutralizing functions of the antibody contribute to its *in vivo* activity. An intravenous challenge allows for rapid systemic delivery of virus. While a single dose of C10 antibody was not able to prevent infection following an intravenous challenge, systemic ZIKV-RNA levels were dramatically reduced, with low levels of ZIKV-RNA detected in some tissues of C10-treated mice. Increasing the antibody dose and/or dosing frequency may be needed to prevent viral spread or clear infection under the extreme conditions of an intravenous challenge. Future studies will indicate the ability of the C10 antibody to reduce ZIKV infection when administered after infection has already been established. Using a true potent ZIKV antibody instead of a cross-reactive antibody may also result in increased efficacy. Recently, a neutralizing antibody isolated from a ZIKV-infected pregnant woman recognizing domain II of the ZIKV envelope dimer was shown to protect Ifnar1−/− mice from a lethal ZIKV challenge (47). Collectively, these results demonstrate that passive administration of ZIKV neutralizing antibodies could be used during future ZIKV outbreaks in high-risk populations to prevent ZIKV transmission. Future studies will also indicate the potential of neutralizing antibodies for the treatment of established ZIKV infection.

## MATERIALS AND METHODS

### Sex as a biological variable

BALB/c (male and female), NOD/SCID (female), and NSG (male and female) mice used in this study were 12–20 weeks of age. NOD/SCID mice were derived by crossing non-obese diabetic (NOD) and SCID mouse strains (48). The NOD strain was derived from Cataract Shinogi mice, an inbred subline of the outbred ICR mouse strain (49). SCID mice arose from a point mutation in chromosome 16 in the CB-17 mouse strain. The C.B-17 strain was derived from the BALB/c strain and has the *Igh-1^b^* allele from the C57BL/Ka strain (50). NSG mice were derived by crossing NOD/SCID mice with C57BL/6.129S4-*Il2rg^tm1Wjl^* mice (51). Similar plasma ZIKV-RNA levels were observed in male and female NSG mice, indicating no notable sex bias in ZIKV replication levels (Fig. S1A). ZIKV-RNA levels were measured in the reproductive tracts of male and female mice during acute and chronic ZIKV infection and following treatment with C10 antibody.

### Virus challenges and administration of DFMA and C10 mAb antibody

Stocks of ZIKV H/PF/2013, SPH2015, PRVABC59, and BeH819015 were prepared as previously described (52). Viral challenges were performed by diluting viral stocks in RPMI medium (Gibco, Gaithersburg, MD). Virus (2.5–1.0 × 10^5^ FFU) was administered intravenously via tail injection (200 µL volume). DFMA was dissolved in DMSO (Fisher Scientific, Hampton, NC) and diluted in PBS (Sigma-Aldrich, St. Louis, MO) before filtration with a 70 µm syringe filter (Corning, NY) and systemically administered via intraperitoneal injection. The monoclonal antibody C10 was prepared using transfected human 293T cells from cloned plasmids as previously described (52). C10 antibody (62.5 µg) was diluted in saline (Hospira, Lake Forest, IL) and administered systemically via intraperitoneal injection (200 µL volume) 18 h before viral challenge.

### Collection and processing of mouse bodily fluids

Mouse peripheral blood and bodily secretions were collected longitudinally for ZIKV-RNA quantification. Peripheral blood was collected into tubes containing an anticoagulant (EDTA solution, Sigma-Aldrich, St. Louis, MO). Plasma was separated by centrifugation. To stimulate salivation, ZIKV-infected mice were administered pilocarpine HCl (Sigma-Aldrich, St. Louis, MO) in sterile PBS (100 µg/100 µL) by intraperitoneal injection essentially as previously described (53). Saliva was collected directly from the mouth with a micropipette. Cervicovaginal secretions were obtained by cervicovaginal lavage with three successive washes of 20 µL sterile PBS using sterile filter micropipette tips inserted less than 3 mm into the vaginal canal. Urine was collected by holding mice above a sterile petri dish and lightly palpating above the bladder to induce urination. The urine was then collected from the petri dish. Cells and debris from bodily fluids were removed by centrifugation.

### CD4^+^ and CD8^+^ T cell depletion and flow cytometric analysis

Mouse CD4^+^ and CD8^+^ T cells were depleted in BALB/c mice by twice weekly intraperitoneal injections of 200 µg anti-mouse CD4 (GK1.5) (Bio X Cell, West Lebanon, NJ) and 200 µg anti-mouse CD8 (2.43) (Bio X Cell, West Lebanon, NJ) diluted in sterile PBS. Immune cell populations present in the peripheral blood and spleen of mice were analyzed by flow cytometry using the following antibodies directed against mCD45 (APC-Cy7, BD Pharmingen, Cat. 559864), mCD3 (PE, BD Pharmingen, Cat. 555275), mCD4 (APC, BD Pharmingen, Cat. 560181), mCD8a (FITC, BD Pharmingen, Cat. 553030), mCD19 (PE-Cy7, BD Pharmingen, Cat. 552854), and mCD11b (PerCP, BD Pharmingen, Cat. 550993). Live cells were distinguished by forward and side scatter profiles. Data were acquired with a BD FACSCanto flow cytometer and analyzed with BD FACS Diva software (version 6.1.3).

### Collection and processing of tissues

Mouse tissues were collected at necropsy following transcardiac perfusion of mice with PBS, essentially as previously described (54–56). Tissues collected for analysis included the spleen, bone marrow, lungs, liver, gastrointestinal tract, brain, and eyes. The reproductive tract was collected from female mice, and the epididymis, testes, prostate, penis, and seminal vesicles (male reproductive tract) were collected from male mice. For ZIKV-RNA analysis, tissues were processed into single-cell suspensions as previously described (53, 56–59). In brief, cells were isolated by forcing tissues through a 70 µm cell strainer (Falcon, Corning, NY) followed by red blood cell lysis if necessary. The liver, lung, female reproductive tract, and penis were digested in an enzyme digest cocktail before filtration. Liver, lung, and brain cells were purified with a Percoll gradient (GE Healthcare, Little Chalfont, UK). The mouse gastrointestinal tract was flushed with PBS and incubated with a dithiothreitol (Fisher Scientific, Hampton, NC) and EDTA solution to isolate cells from the intraepithelial layer and then incubated with elastase (Worthington Biochemical, Lakewood, NJ) and hyaluronidase (Worthington Biochemical, NJ) to isolate the cells from the lamina propria layer (56, 58).

### ZIKV-RNA determinations

RNA was extracted from plasma (40 µL) using the QIAmp Viral RNA kit (Qiagen) (lower level of detection 833 copies/mL). Tissue RNA was extracted using RNeasy mini columns (Qiagen) according to the manufacturer’s protocol, including an optional treatment with RNase-free DNase. ZIKV-RNA levels in the peripheral blood plasma from infected mice were determined using a one-step quantitative real-time PCR (TaqMan RNA-to-CT 1-step kit, Applied Biosystems, Foster City, CA). The sequences of the forward and reverse primers and the TaqMan probe for PCR amplification and detection of ZIKV RNA were 5′-CCGCTGCCCAACACAAG-3′, 5′-CCACTAACGTTCTTTTGCAGACAT-3′, and 5′-FAM- AGCCTACCT/ZEN/TGACAAGCAGTCAGACACACTCAA-Q-3′, respectively (3). ZIKV-RNA was transcribed using a custom-synthesized plasmid (Biomatik) to create a standard curve. ZIKV-RNA levels in samples were quantified by extrapolation from the standard curve. All samples were run and analyzed on an ABI 7500 Fast Real-Time PCR System (Applied Biosystems, Foster City, CA).

### Statistical analysis

All data were graphed and analyzed with GraphPad Prism 10 for statistical analysis. Statistical tests are indicated in the figure legends. Data is presented as the mean ± standard deviation (SD), unless otherwise noted. Plasma and saliva viral load measurements below the limit of detection (833 copies per mL) were graphed at 100 copies per milliliter. Undetectable cell-associated ZIKV-RNA measurements were graphed at 0.5 copies per 100,000 cells.

## Data Availability

All supporting data are available from the corresponding authors upon reasonable request.

## SUPPLEMENTAL MATERIAL

Supplemental material jvi.01147-25-s0001.docx Table S1; Fig. S1 and S2.

ASM does not own the copyrights to Supplemental Material that may be linked to, or accessed through, an article. The authors have granted ASM a non-exclusive, world-wide license to publish the Supplemental Material files. Please contact the corresponding author directly for reuse.
