# Supplementary material for: Envelope-dimer epitope 1 (EDE1) antibody (C10) treatment significantly reduces Zika virus replication in the male and female reproductive tracts
Source: J Virol. 2025 Aug 18;99(9):e01147-25. doi: 10.1128/jvi.01147-25 (PMC12456144; doi:10.1128/jvi.01147-25)
Supplement: Supplemental material — Table S1; Fig. S1 and S2. [file jvi.01147-25-s0001.docx]

**Table S1.** **ZIKV-RNA is shed into the urine and cervicovaginal secretions of ZIKV-infected immune deficient mice.**

| Week Post Exposure |  | CVS | Urine |
| --- | --- | --- | --- |
| 2 |  | N.A. | 0/1 |
| 3 |  | 2/3 | 2/2 |
| 5 |  | 3/3 | 1/1 |
| 6 |  | 3/3 | 1/2 |
| 8 |  | 1/1 | 1/1 |
| 9 |  | N.A. | 0/3 |

^#^Bodily secretions were sampled from ZIKV infected mice longitudinally and ZIKV-RNA was analyzed by real time-PCR. Results indicate the proportion of mice positive for ZIKV-RNA in cervicovaginal secretions (CVS) and urine at each time point. N.A. indicates samples not analyzed.

**Supplemental Figures**

**
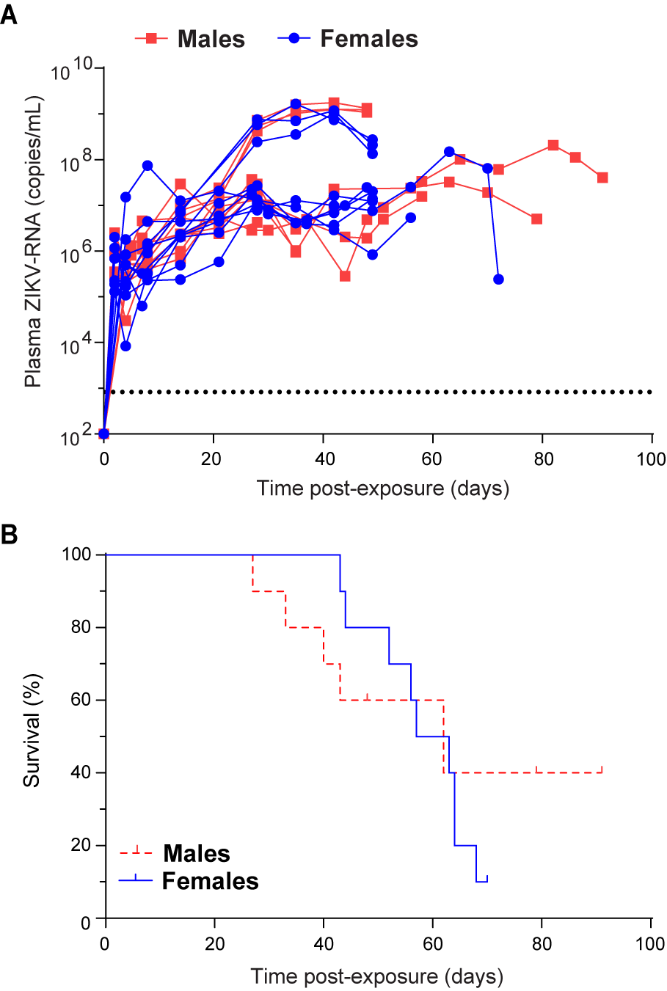
**

**Fig S1. Analysis of plasma ZIKV-RNA levels and survival in male and female mice.** (A) Plasma ZIKV-RNA levels in male (n=10; red) and female (n=10; blue) NSG mice intravenously exposed to ZIKV H/PF/2013 (0.25 – 1 x 10^6^ FFU). Dashed line: limit of detection (833 copies/mL). (B) Kaplan-Meier graph comparing post-exposure survival of male (n = 10; red line) and female (n = 10; blue line) NSG mice exposed to ZIKV H/PF/2013 (0.25 – 1 x 10^6^ FFU). Mantel-Cox log rank test, p = 0.8317.

**
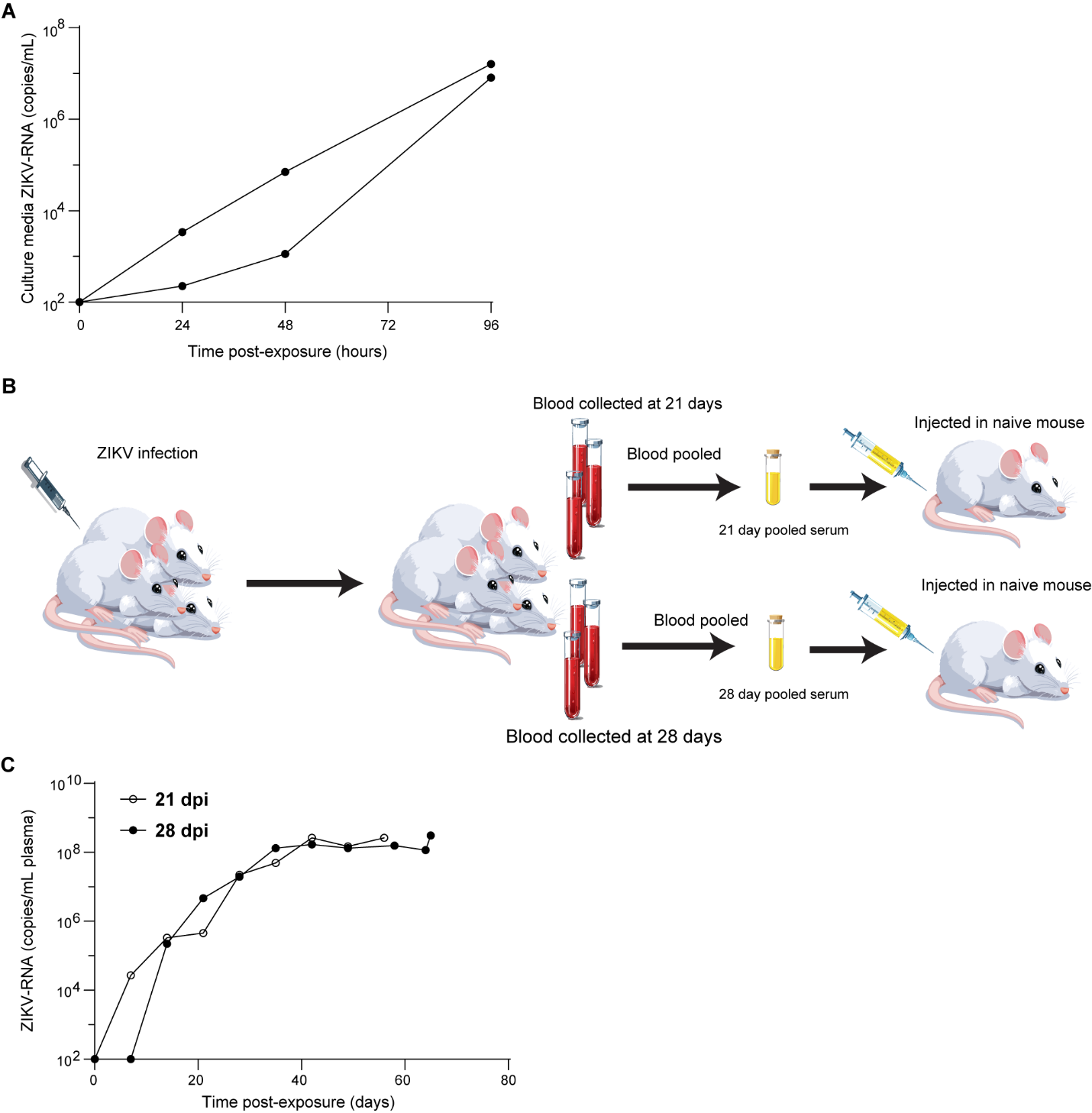
**

**Fig S2. ZIKV from serum of infected mice efficiently replicates *in vitro* and *in vivo*.** (A) Serum (3µL) from ZIKV-infected mice was added to VERO cells, and ZIKV-RNA levels were quantified in culture medium 24h, 48h, and 96h later. (B) Experimental design. Three NSG mice were exposed intravenously to ZIKV H/PF/2013. At 21 days and 28 days post-exposure, 20µL serum was collected from each mouse and pooled before being intravenously injected into a naïve NSG mouse (n=1 mouse/time point). (C) Plasma ZIKV-RNA levels were measured over time in recipient mice. Limit of detection (833 copies/mL plasma).
